# Influence of the geometry of nanostructured hydroxyapatite and alginate composites in the initial phase of bone repair^1^

**DOI:** 10.1590/s0102-8650201900203

**Published:** 2019-02-28

**Authors:** George Gonçalves dos Santos, Luisa Queiroz Vasconcelos, Suelen Cristina da Silva Poy, Renata dos Santos Almeida, Aryon de Almeida Barbosa, Silvia Rachel de Albuquerque Santos, Alexandre Malta Rossi, Fúlvio Borges Miguel, Fabiana Paim Rosa

**Affiliations:** I Master, Fellow PhD degree, Postgraduate Program in Interactive Processes of Organs and Systems, Health Sciences Institute, Universidade Federal da Bahia (UFBA), Salvador-BA, Brazil. Technical procedures, manuscript writing.; II Master, Fellow PhD degree, Postgraduate Program in Interactive Processes of Organs and Systems, Health Sciences Institute, UFBA, Salvador-BA, Brazil. Technical procedures.; III Master, Faculdade Maria Milza (FAMAM), Mangabeira-BA, Brazil. Technical procedures, histological examinations.; IV PhD, Postgraduate Program in Interactive Processes of Organs and Systems, Health Sciences Institute, UFBA, Salvador-BA, Brazil. Histomorphometric examinations.; V PhD, Researcher Collaborator, Laboratory of Tissue Bioengineering and Biomaterials (LBTB), Health Sciences Institute, UFBA, Salvador-BA, Brazil. Histopathological examinations.; VI Chemistry, LABIOMAT, Brazilian Center for Physics Research, CBPF, Rio de Janeiro-RJ, Brazil. Analysis, synthesis and physicochemical characterization of biomaterials.; VII PhD, LABIOMAT, Brazilian Center for Physics Research, CBPF, Rio de Janeiro-RJ, Brazil. Analysis, synthesis and physicochemical characterization of biomaterials.; VIII PhD, Associate Professor, Health Sciences Center, Universidade Federal do Recôncavo da Bahia (UFRB), Santo Antonio de Jesus-BA, Brazil. Technical procedures, critical revision.; IX PhD, Associate Professor, Health Sciences Institute, UFBA, Salvador-BA, Brazil. Conception of the study, critical revision.

**Keywords:** Durapatite, Alginates, Polymers, Microspheres, Rats

## Abstract

**Purpose:**

To analyze, histomorphologically, the influence of the geometry of nanostructured hydroxyapatite and alginate (HAn/Alg) composites in the initial phase of the bone repair.

**Methods:**

Fifteen rats were distributed to three groups: MiHA - bone defect filled with HAn/Alg microspheres; GrHA - bone defect filled with HAn/Alg granules; and DV - empty bone defect; evaluated after 15 days postoperatively. The experimental surgical model was the critical bone defect, ≅8.5 mm, in rat calvaria. After euthanasia the specimens were embedded in paraffin and stained with hematoxylin and eosin, picrosirius and Masson-Goldner’s trichrome.

**Results:**

The histomorphologic analysis showed, in the MiHA, deposition of osteoid matrix within some microspheres and circumjacent to the others, near the bone edges. In GrHA, the deposition of this matrix was scarce inside and adjacent to the granules. In these two groups, chronic granulomatous inflammation was noted, more evident in GrHA. In the DV, it was observed bone neoformation restricted to the bone edges and formation of connective tissue with reduced thickness in relation to the bone edges, throughout the defect.

**Conclusion:**

The geometry of the biomaterials was determinant in the tissue response, since the microspheres showed more favorable to the bone regeneration in relation to the granules.

## Introduction

 Researchers of the bone tissue bioengineering have sought to develop ideal conditions for the repair and/or replacement of damaged or lost tissue through the use of cellular elements, growth factors, regenerative techniques and biomaterials, in order to provide the *scaffold* and essential requirements for tissue neoformation^1^ ^-^ ^3^. 

 The biomaterials can be synthesized from different substrates and processed in different forms of presentation, namely: fiber, membrane, gel, powder, among others; and different geometries such as plates, cylinders, microspheres and granules. Microspheres have attracted great scientific interest due, in particular, to their ability to promote the formation of interstices between them, which allows cell migration, adhesion, proliferation and differentiation, mainly, mesenchymal and osteoprogenitoring, liberation of growth factors, angiogenesis, diffusion of nutrients and new extracellular matrix (ECM) synthesis^4^. By turn the granules, in addition to having the aforementioned properties, are widely used in clinical practice in the filling of defects and tissue lesions with irregular shapes. Both microspheres and granules can be applied through injectable systems in minimally invasive surgical procedures^5^. 

 Among the substrates most used in the synthesis of biomaterials with the geometry of microspheres and granules, bioceramics based on calcium phosphate (CaP) stand out, mainly, the HA due to its biocompatibility, osteoconduction and bioactivity^6^. However, in spite of these fundamental properties, in the biological interaction with the host, the HA presents a low *in vivo* degradation rate which, in some applications, may limit its use^7^. 

 In seeking to improve the physicochemical characteristics of HA, researchers have developed this material on a nanometer scale, considering that the HA nanostructured crystals (HAn) have higher biodegradation due to the smaller size of its particles and larger surface area exposed to the biological environment, which accelerates the speed of formation and growth of the biologically active apatite layer^7^ ^,^ ^8^. Another way to improve bioceramics is to associate them with natural or synthetic polymers to produce biomaterials of the type composites^2^ ^,^ ^9^. These materials have, in the same *scaffold*, physical-chemical properties of the ceramic and polymer, which are improved in relation to the materials when used in an individual way and mimetize the inorganic and organic phases of the natural bone^10^ ^-^ ^12^. 

 In this perspective, the alginate is a natural polymer widely used, since this material can alter the crystallinity, solubility, network parameters, thermal stability, surface reactivity, bioactivity and adsorption properties of the HA structure^9^ ^,^ ^13^ ^,^ ^14^. Therefore, the physicochemical characteristics of the composites vary according to the polymer and its percentage used during the synthesis, as well as the processing of the sample and the final geometry of the biomaterial produced. Thus, these biomaterials, especially in the form of microspheres and granules are configured as a promising alternative to bone substitution, particularly in situations where damage and/or trauma reach critical dimensions which preclude spontaneous bone regeneration and hinders the functional property or aesthetic of the affected^15^ ^,^ ^16^.

 Given the above, parallel to the need, in worldwide level, to develop new biomaterials, with national technology and affordable cost, most versatile, and promising biological properties for use, especially in cases of extensive bone loss, the present study aims to analyze the influence of the geometry of HAn and alginate composites in the initial phase of bone repair.

## Methods

 Surgical procedures were performed according to the Ethical Standards of Researches in Animals (Law No. 11,794 of 2008); the National Biosafety Standards and the National Health Institute Guidelines for the Care and Use of Laboratory Animals (NIH Publication No. 85-23, Rev. 1985), upon approval of the Ethics Committee on the Use of Animals of the Health Sciences Institute, Universidade Federal da Bahia, protocol 038/2012. 

###  Biomaterials 

#### Synthesis and processing of the HAn and alginate microspheres and granules

 The biomaterials were synthesized, processed and characterized in the LABIOMAT (Brazilian Center for Physical Research, CBPF, Rio de Janeiro, Brazil). The synthesis was realized by mixing a solution of ammonium hydrogenphosphate [(NH_4_) _2_HPO_4_], maintained at pH 11, to a solution of calcium nitrate tetrahydrate [Ca(NO_3_)_2_.4H_2_O] under constant stirring, under synthesis temperature of 90^o^C. 

 The precipitate resulting was filtered and washed until the pH of the wash water was 7. Soon after, the solid obtained was dried by freeze-drying for 24h and then separated using sieves with a desired *mesh* aperture. 15g of the obtained solid were weighed into *becker* and added to a 1.5% w/v solution of sodium alginate, vigorously mixed until obtain a homogeneous paste.

**Figure 1:**
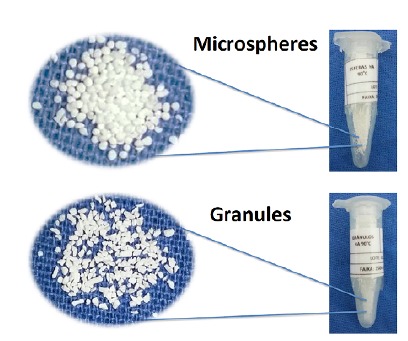
Macroscopic aspect of biomaterials.

 To obtain the microspheres, this paste was extruded with the use of a syringe in 0.15M calcium chloride solution at room temperature. Finally, the microspheres were washed and dried in a glass-house at 50º C and then submitted to the sieve with a granulometric range of 250-425 μm. In order to obtain the granules, the obtained paste was dried in a glass-house and then crushed and sieved in the granulometric range of 250-425 μm. The biomaterial samples were conditioned in *eppendorf* tubes, properly identified, and sterilized by gamma rays (Fig. 1). Each aliquot was used to fill the bone defect of, approximately, four animals. 

###  Physical-chemical characterization of HAn powders 

#### Superficial area

 The characterization of the surface area by the BET method (ASAP 2020 *- Micromeritics* ^®^) showed that the HA evaluated in our study has a surface area of 35.9501 m^2^/g, characteristic of manometric biomaterials. 

#### Chemical analysis

 The chemical analysis of the X-ray fluorescence (XRF) (PW2400-*Philips* ^®^) demonstrated that HA evaluated in this work presents Ca/P molar ratio of 1.67 (Table 1).

**Table 1:** XRF analysis.

Sample	Ca %	mol do Ca	P %	mol do P	Ratio Ca/P
HA	35.70	0.8908	16.40	0.52948	1.6823
HA	36.00	0.8982	16.60	0.53593	1.6760
HA	37.12	0.9262	17.20	0.55530	1.6679
				**Mean**	**1.6754**

Ca % = Percent of Calcium. mol of Ca = Calcium Concentration. P % = Percent of Phosphate. mol of P = Phosphate Concentration. Ratio Ca/P = ratio of calcium and phosphate.

#### X-ray diffraction (X-RD) analysis

**Figure 2:**
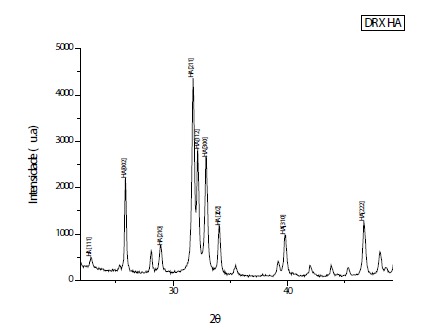
Peaks corresponding to the crystallinity of a standard HA are noted.

 The analysis of X-ray diffraction was performed using the high-resolution diffractometer HZG4 (*Zeiss* ^®^) with CuKa radiation (l = 1.5418 Å) and angular scanning of 10-80^o^(2ɵ), with step of 0.05/s, time 160 seconds, with reference to the standard PCPDFWIN 09.0432 (International Centre for Diffraction Data - ICDD)^17^ ^,^ ^18^. The diffractogram evidenced peaks corresponding to the crystalline profile of a standard HA (Fig. 2). 

#### Fourier Transform Infrared Spectroscopy (FTIR)

**Figure 3:**
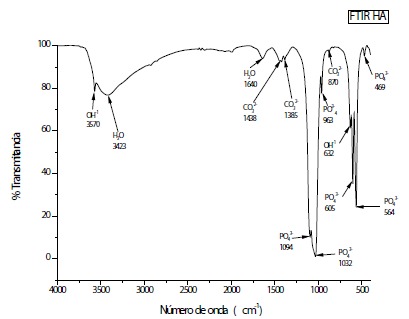
Vibrational spectra of Infrared of the sample prepared at 90º C. It is observed that the spectra revealed characteristic bands of HA for phosphate (PO_4_^3-^), water (H_2_O) and hydroxyl (OH^1-^).

 The FTIR analysis evidenced vibrational spectra corresponding to those of a standard HA^17^ ^,^ ^18^, it is noted in the regions of 3430 and 1646 cm^-1^ intense and wide water bands. In the regions 1462 to 1414 cm^-1^ are found the characteristic bands of the carbonate ions, showing that the alginate is present in the sample. The other bands observed in 1038, 961, 602 and 560 cm^-1^ are characteristic of phosphate ions present in HA. Even the sample containing a large amount of water, it was possible to identify the hydroxyl bands in 3570 and 635 cm^-1^ (Fig. 3). 

###  Experimental phase 

 Fifteen male *wistar* albino adult rats with body weight between 350 and 400 g were randomly distributed to compose three experimental groups, with 5 animals in each group: MiHA - bone defect filled with HAn microspheres and alginate; GrHA - bone defect filled with HAn granules and alginate; DV - bone defect without implantation of biomaterial.

###  Surgical procedure 

**Figure 4:**
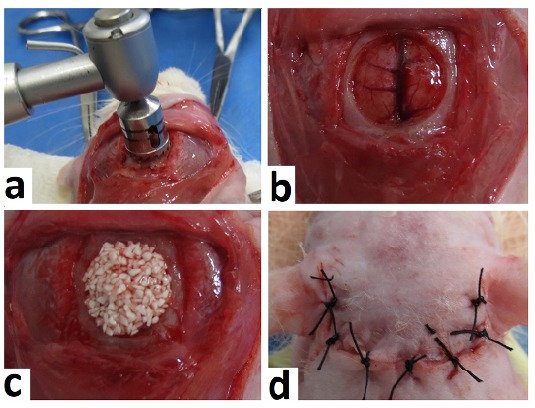
Steps of the surgical procedure. **a**) Removal of the bone fragment with trephine drill. **b**) Critical bone defect. **c**) Implanted Biomaterial. **d**) Tissue flap repositioned and sutured.

 The surgical technique used was the same described by Miguel *et al.* ^19^. However, in the present study, the 8.0 mm trephine drill used in the confection of the critical bone defect was with a diameter of ≅8.5 mm and ≅0.8 mm of thickness. After removal of the bone fragment, it was procedered the implantation of the biomaterials, according to each experimental group. In the control group (DV) the bone defect remained empty, without biomaterial. Finally, the tissue flap was repositioned and sutured with simple points (Fig. 4).

###  Histological processing and histomorphological analysis 

 After 15 days post surgery, the animals were euthanized and the calvaria removed. The obtained specimens were fixed formaldehyde 4% for 48 hours, included in paraffin and subsequently cut to 5μm of thickness and stained with hematoxylin-eosin (HE), picrosirius-red (PIFG) and Masson-Goldner trichrome (GOLD) and analyzed under an optical microscope (DM1000 *-* *Leica* ^®^). The images were captured using a digital camera (DFC310FX *-* *Leica* ^®^). For morphometric analyses, the Leica Application Suite (version 4.12 - *Leica* ^®^) and optical microscope (DM6 B *-* *Leica* ^®^) were used. To compare the differences between the groups the Kruskal-Wallis test was applied using the software SPSS version 20.0 (*IBM* *SPSS* ^®^), at a 5% level of significance (*p*≤0.05).

## Results

**Figure 5:**
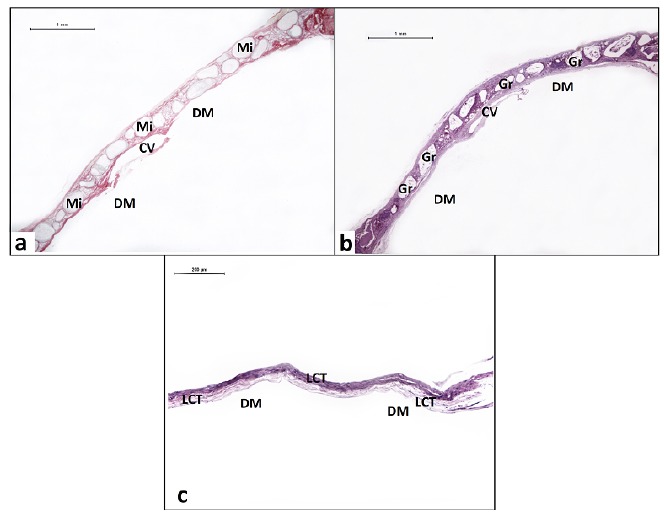
**a**) MiHA - Microspheres distributed along the bone defect. PIFG. Bar: 1 mm. **b**) GrHA - Granules arranged along the bone defect. HE. 1 mm bar. **c**) DV - Deposition of conjunctive tissue throughout the bone defect. HE. Bar: 200 μm. Microspheres (Mi), granules (Gr), loose connective tissue (LCT), central vein (CV) and dura mater region (DM).

 The histomorphologic analysis evidenced neoformation of osteoid matrix restricted to the borders of the bone defect with formation of fibrous connective tissue in the remaining area, in all experimental groups. When compared to the bone edge, the tissue thickness produced in the defect region remained proportional in the groups with implantation of microspheres and granules, and significantly reduced in the group without implantation of biomaterials (Fig. 5). 

**Figure 6:**
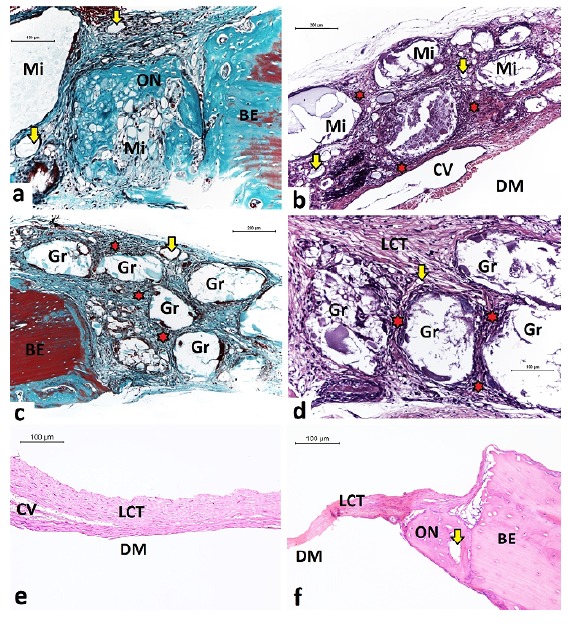
**a**) MiHA - Neoformation osteoid within the microsphere and discrete circumferential chronic granulomatous inflammation. GOLD. Bar: 100 μm. **b**) MiHA - Microspheres partially degraded and tissue neoformation within the particles. HE. Bar: 200 μm. **c**) GrHA - integrity of granules and scarce neoformation osteoid restricted to the bone edges. GOLD. Bar: 200 μm. **d**) GrHA - Intense chronic granulomatous inflammation surrounding the granules. HE. Bar: 100 μm. **e**) DV - Tissue thickness significantly reduced along the central region of bone defect. HE. Bar: 100 μm. **f**) DV - Tissue thickness significantly reduced to the bone edges. HE. Bar: 100 μm. Microspheres (Mi), granules (Gr), loose conjunctive tissue (LCT), osteoid neoformation (ON), bone edge (BE), blood capillary (yellow arrows), chronic granulomatous inflammation (red stars), central vein (CV), dura mater region (DM).

 In the MiHA, the biomaterials were arranged in monolayer, with small variation of microspheres size, throughout the extent of the bone defect. The majority remained intact and some presented partial and/or total fragmentation with neoformation of osteoid matrix within the *scaffold*. In this group, it was observed the presence of mononuclear inflammatory cells and multinucleated giant cells, characteristic of chronic granulomatous inflammatory response, discreetly noticed around the microspheres, especially those located at the periphery of the bone defect. In GrHA, the granules were distributed in mono and multilayer, throughout the extent of the bone defect. In this group the chronic granulomatous inflammatory reaction observed was more evident than in the MiHA. Most of the granules remained intact, while others presented partial fragmentation, less accentuated in relation to the microspheres, without osteoid neoformation inside of the biomaterials. In both groups with implantation of biomaterials, it was noticed abundant proliferation of blood capillaries around the particles (Fig. 6). 

 The histomorphometric analysis measured the percentage of mineralized linear extension (Table 2). The Kruskal-Wallis test demonstrated statistically significant difference between the 3 groups (MiHA x GrHA x DV - *p*=0.007) and between the experimental and control groups - MiHA x DV (*p*=0.010) and GrHA x DV (*p*=0.046). In the groups which the biomaterials were implanted the Kruskal-Wallis test did not demonstrate statistically significant difference- MiHA x GrHA (*p*=1.00).

**Table 2:** Histomorphometry of linear extension and mineralized linear extension.

Groups	LE (mm) Mean(SD)	MLE (mm) Mean(SD)	MLE (%) Mean(SD)
MiHA	7.07(0.30)	1.87(0.69)	26.46(9.78)
GrHA	7.28(0.45)	1.46(0.73)	19.73(9,17)
DV	8.18(0.11)	0.76(0.09)	9.34(1.05)

LE = Linear extension. MLE = Mineralized Linear Extension. SD = Standard Deviation.

## Discussion 

 The bone repair mechanism is a dynamic, temporal and complex phenomenon that depends on the presence of a three-dimensional scaffold and on the manner in which the bone, undifferentiated mesenchymal and endothelial cells interact with each other and with the microenvironment around them. Under the physiological conditions, this event is consolidated by regeneration. On the other hand, when the tissue loss presents critical dimensions, extension and morphology, there is impairment of this mechanism and the tissue repair occurs by fibrosis^19^ ^,^ ^20^. Under these conditions, bone regeneration becomes limited, as observed in the control group (DV) of our study and in other studies^19^ ^-^ ^22^. These results validate the simulation of extensive bone loss as it occurs in cases of congenital pathologies, extensive surgical resections, trauma and severe inflammatory diseases. In view of these inhospitable conditions, it is evidenced the need for the use of bone substitutes that make feasible the complete tissue regeneration.

 The spatial arrangement of the biomaterials and the neoformed tissues between and around the particles, in the two implanted groups showed that the microspheres and granules acted as scaffolds suitable to applications as fillers materials. However, in view of the almost complete reduction of the neoformed interstitium between the granules, it is noted that the spatial arrangement of these biomaterials, similar to the mosaic, interfered in the migration of the cells between the particles during bone repair. These findings were consonant those were evidenced by Ribeiro *et al.* ^22^, who evaluated granules of HA and alginate in the same biological point.

 The evident presence of neoformed blood vessels surrounding the biomaterials demonstrates that the materials evaluated were biocompatible and osteoconductive, independent of the geometric shape, and provided a structure favorable to migration, adhesion and proliferation cellular, the release of growth factors, angiogenesis and tissue neoformation^4^ ^,^ ^12^ ^,^ ^23^. Thus, the neoformed osteoid matrix was similarly consolidated in both groups with implantation of biomaterials (*p*>0.05). 

 The partial biodegradation of the biomaterials evaluated in this study, most noticeable in the MiHA, demonstrated the influence of alginate on the materials that, when coming into contact with fluids and living tissues, was dissolved by local enzymes and biorreabsorbed. In this way, it induced the gradual release of the inorganic components of the composite, mainly ions of Ca and PO_4_, contained in the crystals of HA. These results contradict those observed by Barreto^24^, in which the microspheres of HA and alginate did not undergo evident biodegradation at the same biological point. This occurred due to the removal of the polymer from the structure of the microspheres during the calcination process. This finding can be attributed, in sum, to the size of the microspheres used by Barreto^24^ in relation to those used in our experiment, 400-600 μm and 250-425 μm, respectively, because the smaller the particle of the biomaterial, the larger the superficial area of the material exposed to the biological environment. In view of these findings, it is noted that the calcination and sintering processes increase crystallinity, since it favors crystal fusion, aggregation and growth particle, making them resistant to biodegradation^24^. Corroborating with this analysis, in the study by Paula *et al.* ^25^, non calcined microspheres with 400μm showed partial biodegradation with deposition of collagen fibers inside the particles. Conversely, in the study realized by Rossi *et al.* ^26^. the sintered HA microspheres, with the same size as those used in our study, did not present expressive biodegradation, at the same biological point. 

 One of the technological innovations presented by the biomaterials evaluated in this study, in both geometric shape, was the conception of the HA at nanometric scale. As observed in the MiHA, according to Valenzuela *et al.* ^8^, the nanostructured HA crystals tend to dissolve (biodegrade) faster because of the smaller particle size and the larger surface area exposed to the biological environment.

 In our study, the chronic granulomatous inflammation, noted in both groups in which biomaterials were implanted, was compatible with that expected every time a biomaterial is implanted in the living organism^27^ ^,^ ^28^. This finding proves the biocompatibility of the biomaterials, since there was no rejection by the organism, characterized by acute exacerbated inflammation^27^ ^,^ ^28^. This potentiality can be attributed, mainly, to the physical-chemical composition of the materials that mimic the inorganic and organic phases of the natural bone tissue, by HAn and alginate, respectively. On the other hand, the more evident chronic granulomatous inflammation in the GrHA, in comparison to the MiHA, reveals that the irregular surface of the granules modulated the cellular response in the interstice between the particles of the biomaterial^27^ ^,^ ^28^. These findings reveal that the geometry of the biomaterials interferes directly in the tissue response to the presence of the particles^5^ ^,^ ^29^.

 In the study by Ribeiro *et al.* ^22^ the microspheres acted better as filling scaffold and the granules presented superior osteoconductive potential, contrasting our results. It should be emphasized which, in that study, the biomaterials had a diameter of 425-600 μm; the granules contained 1% of alginate; and the biomaterials were synthesized by another way. In our experiment, the percentage of alginate used was 1.5% and diameter of 250-425 μm, which may have influenced the tissue response to granules^22^. 

 Considering that the results of this study are related to the initial phase of the bone repair mechanism (15 days) and can influence, significantly, subsequent cellular events, it becomes pressing the need to develop new studies to observe this response in the long term.

## Conclusions

 In the initial phase of bone repair, the geometry of the biomaterials influenced the tissue response to the implantation of HAn and alginate composites. Both biomaterials exhibited neoformation of osteoid matrix, although the microspheres exhibited histological characteristics more favorable to bone regeneration than granules.
